# Dietary Habits of Students Enrolled in Faculties of Health Sciences: A Cross-sectional Study

**DOI:** 10.7759/cureus.6012

**Published:** 2019-10-28

**Authors:** Sarah AlJohani, Mahmoud Salam, Ala'a BaniMustafa, Abdul Rehman Z Zaidi, Abdulaziz A Aljohani, Adel Almutairi, Majed A AlJohani, Mohammed AlSheef

**Affiliations:** 1 Miscellaneous, King Saud bin Abdulaziz University for Health Sciences, Riyadh, SAU; 2 Nursing, Hariri School of Nursing, American University of Beirut, Beirut, LBN; 3 King Abdullah International Medical Research Center (KAIMRC), King Saud bin Abdulaziz University for Health Sciences, Riyadh, SAU; 4 Internal Medicine, King Fahad Medical City, Riyadh, SAU; 5 Medicine, King Saud University, Riyadh, SAU; 6 Medicine, King Fahad Medical City, Riyadh, SAU

**Keywords:** medical education, healthy, diet, unhealthy, health sciences, students, dietary habits, lifestyle, health care professionals

## Abstract

Introduction

It is often presumed that students of health sciences are more vigilant about their diet. This study assessed the prevalence of unhealthy dietary habits and identified its associated factors among students enrolled at a large university for health sciences in the Middle East.

Methods

A cross-sectional study, using a set of pre-validated and anonymous dietary tools, was conducted in 2018. The self-reported students’ characteristics and prevalence of 10 unhealthy dietary habits were collected.

Results

Males were significantly more likely have irregular meal times (β = 0.425, adjusted [adj.] odds ratio [OR] = 1.5) and insufficient seafood consumption (β = 0.55, adj. OR = 1.7) compared to females, adj. *P *= 0.046 and adj. *P *= 0.012, respectively. Students in their third year and above (*β *= 0.857, adj. OR = 2.2) reported more insufficient water intake compared to students in the first and second years, adjusted *P *= 0.003. Obesity in students was a significant associated factor with fast food consumption (β = 0.48, adj. OR = 1.8), night-eating habits (β = 0.27, adj. OR = 1.3) and skipping meals (β = 0.41, adj. OR = 1.5) compared to normal weight students, adjusted *P *= 0.002, adj. *P *= 0.004, and adj. *P *= 0.003, respectively.

Conclusions

Compliance with healthy dietary habits among students was less than optimal. Special consideration should be paid to gender and obesity that have been associated with insufficient water intake, irregular meal times, skipping meals, night-eating habits, and fast food consumption.

## Introduction

Assessing whether a dietary habit is healthy or not and generating plans for weight control and disease prevention have been extensively presented in the literature. Various dietary assessment tools have been constructed and validated on various age groups and age/diseases specific populations. For instance, the Nutrition Literacy Assessment Instrument (NLit) demonstrated substantial factor validity and test-retest reliability in adult primary care patients, whereas a brief folate-specific food frequency questionnaire for Central and Eastern European population was considered as a valid tool for the assessment of folate intake in young women [1-2].

A self-reported dietary profile of any individual ought to be analyzed and evaluated at its specific components. For instance, a comprehensive dietary profile assessment should include questioning the individual on the intake of sugary drinks, sweetened foods, fruits/vegetables, and so on. A scoring system that scores, quantifies, and accumulates various scores of dietary habits into one summated score and sets a certain cut-off point might defer the attention of nutritionists from specific problematic dietary habits. These tools might not be fully comprehensive to question subjects about other dietary patterns such as the regularity of meal times, skipping meals, or even dehydration. Therefore, analyzing each dietary habit and evaluating it against rigorous age/disease-specific nutritional guidelines remains the most accurate method to meticulously determine the prevalence of unhealthy dietary habits.

A poor dietary habit might influence the academic performance of students, whereas a weak academic performance might stress students out, leading them to adopt unhealthy dietary behaviors, as a method to overcome their frustration. For instance, some students who are under academic stressors might tend to eat more or vice versa. Students enrolled in health-related faculties such as medicine, nursing, pharmacy, and others are expected to be extra vigilant about their dietary habits, compared to students of other faculties. The level of knowledge on ideal eating habits and disease prevention is expected to be higher among students of health faculties, so their compliance with a healthy diet is anticipated to be higher too. We investigated the dietary habits of students enrolled in the faculties of health sciences at a large university in Riyadh, Saudi Arabia.

## Materials and methods

This was a cross-sectional study conducted among students enrolled at King Saud bin Abdulaziz University for Health Sciences (KSAU-HS), Riyadh, Saudi Arabia. This study aimed to assess the prevalence of a number of unhealthy dietary habits of college students in a large Middle Eastern university for health sciences. Approval was obtained from the Institutional Review Board (RC18/146) at King Fahad Medical City and the Saudi Ministry of National Guard-Health Affairs (SMNG-HA). Inaugurated in 2005, under the directory of SMNG-HA, KSAU-HS is a large-scale university with its main campus located in Riyadh (capital of Saudi Arabia) and two additional campuses in Eastern and Western regions of the Kingdom. KSAU-HS joins seven colleges, Colleges of Medicine, Dentistry, Pharmacy, Public Health and Health Informatics, Applied Medical Sciences, Nursing, and Science & Health Professions. The number of students enrolled per each academic year in both undergraduate and postgraduate studies exceeds 6,000.

By convenience, a pre-validated English language survey was distributed among students in the academic year 2017-2018. Written informed consent was sought with no student identifiers collected. A team of data collectors included a student from the college of pharmacy and a research coordinator. The data collection tool assessed the students’ characteristics, which are gender, age (years), BMI (kg/m^2^) categorized into underweight, normal weight, pre-obese, and obese based on World Health Organization (WHO) and locally conducted study [3]. Students were reminded that their participation and self-reported answers will be confidential and only for research purposes. Respondents included students from Colleges of Medicine (*n *= 116, 31.2%), Dentistry (*n *= 49, 13.2%), Pharmacy (*n *= 47, 12.6%), Applied Medical Sciences (*n *= 98, 26.3%), Nursing, and Science & Health Professions (*n *= 62, 16.7%).

A total of 10 dietary habits were evaluated as healthy or unhealthy based on published literature. Students were asked to describe their routine and standard dietary habits in the past two weeks. Drinking at least one sugar-sweetened beverage (200-250 ml), such as canned juices and sodas, on a daily basis was accounted for as an unhealthy dietary habit [4]. Students who drank less than two liters of water daily (gender adjusted) were classified as having an unhealthy habit, too [5]. The same applied to students who did not consume 400 g of fruits/vegetables on a daily basis and those who consumed fast food meals at least once per week [6-7]. Students who have irregular timings of meals, skipping meals, and night-eating habits were classified as unhealthy dietary habits [8-10]. Consuming unhealthy snacks between meals on a daily basis and drinking at least one energy drink on a weekly basis were classified as unhealthy [11-12]. Eating at least one meal (227 g) of seafood on a weekly basis was accounted for as a healthy dietary habit [13]. 

The statistical software SPSS version 25.0 has been used for data analysis. Descriptive statistics of categorical variables such as student characteristics and dietary habits were presented in frequency (n) and percentage (%), while continuous variables were presented in mean (x̄) and standard deviation (±SD). Analytical statistics included Pearson’s Chi-square (χ^2^) for categorical outcomes. Outliers were excluded, while missing outcome variables were replaced by the mean of sample outcomes. Each dietary habit was accounted for as an individual outcome in 10 binary logistic regression models to identify whether gender, academic year, or BMI were significantly associated factors. Statistical significance was set at *P *< 0.05.

## Results

Sample and outcome characteristics

The sample comprised 392/430 (91%) students who agreed to participate and submitted a complete survey, with a male-to-female ratio (1:1.3) and a mean of age 21 ± 2 years. A very less number of students were married (8; 2%), and only 68 (17.3%) had chronic diseases such as asthma, diabetes type I and/or hypertension. Their average BMI was 23 ± 5, with 225 (57%) classified under the normal weight category and 117 (28.8%) as pre-obese/obese. Those in their first two academic years were 146 (37%), while those in their third & above academic years were 246 (63%). Almost 117 (30%) were in the college of medicine, 109 (28%) in applied medical sciences, 67 (17%) in dentistry, and 99 (25%) in two other faculties. The majority of the sample were in pursuit of a bachelor's degree 380 (97%; Table 1). 

**Table 1:** Distribution of basic characteristics among students n: frequency, %: percentage, x̄: mean, SD: standard deviation

	n (%) 392 (100.0)
Gender	
Females	224 (57.1)
Males	168 (42.9)
Age category (years)	
(x̄ ± SD)	21±2.2
BMI	
Underweight	50 (12.8)
Normal weight	225 (57.4)
Pre-obesity	78 (19.9)
Obesity	39 (9.9)
(x̄ ± SD)	23.4±5.4
Chronic disease	
Yes	68 (17.3)
No	324 (82.7)
Academic year	
First & second	146 (37.2)
Third & above	246 (62.8)

**Figure 1:**
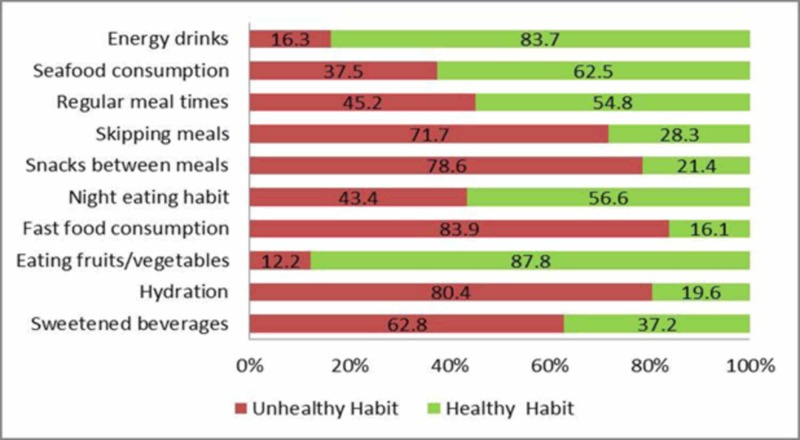
Distribution of unhealthy and healthy dietary habits among students

The prevalence of 10 unhealthy dietary habits is presented in Figure 1.

Unhealthy dietary habits found were drinking sugar-sweetened beverages (246; 62.8%), insufficient daily amounts of water or hydration (315; 80.4%), and consuming energy drinks (64; 16.3%). Almost 177 (45%) had irregular meal times, 308 (78.6%) consumed unhealthy snacks between meals, and 281 (72%) skipped meals, while 170 (43.4%) had night-eating habits, all accounted as unhealthy dietary habits. Other unhealthy habits were prevalent in regard to insufficient eating of fruits/vegetables 48 (12.2%), inadequate seafood consumption 147 (37.5%), and fast food consumption 329 (83.9%; as shown in Figure 1). 

The prevalence of unhealthy dietary habits across sample characteristics is tabulated in Table 2.

**Table 2:** Unhealthy dietary habits across sample characteristics χ2: Pearson’s chi-square, t: Student’s t-test; F: one-way ANOVA; P-value: statistically significant at <0.05, %: percentage, ANOVA: analysis of variance; df: degree of freedom, n: frequency, x: mean, SD: standard deviation

	Gender		Academic year		BMI (category)	
	Females	Males	First & second	Third & above	Underweight/ Normal	Pre-obese/ obese
Sweetened beverages	137(61.2)	109(64.9)	92(63.0)	154(62.6)	164(59.6)	82(70.1)
	χ2 = 0.568, P = 0.451		χ2 = 0.007, P = 0.935		χ2 = 3.834, P = 0.050	
Insufficient hydration	194(86.6)	121(72.0)	109(74.7)	206(83.7)	225(81.8)	90(76.9)
	χ2 = 12.935, P<0.01*		χ2 = 4.788, P = 0.029*		χ2 = 1.246, P = 0.264	
Drinking energy drinks	35(15.6)	29(17.3)	27(18.5)	37(15.0)	45(16.4)	19(16.2)
	χ2 = 0.188, P = 0.664		χ2 = 0.799, P = 0.371		χ2 = 0.001, P = 0.976	
Irregular meal times	114(50.9)	101(60.1)	81(55.5)	134(54.5)	158(57.5)	57(48.7)
	χ2 = 3.300, P = 0.069		χ2 = 0.038, P = 0.846		χ2 = 2.530, P = 0.112	
Snacks between meals	183(81.7)	125(74.4)	122(83.6)	186(75.6)	217(78.9)	91(77.8)
	χ2 = 3.032, P = 0.082		χ2 = 3.441, P = 0.064		χ2 = 0.062, P = 0.803	
Skipping meals	174(77.7)	107(63.7)	111(76.0)	170(69.1)	187(68.0)	94(80.3)
	χ2 = 9.254, P = 0.002*		χ2 = 2.163, P = 0.141		χ2 = 6.159, P = 0.013*	
Night eating habit	91(40.6)	79(47.0)	66(45.2)	104(42.3)	106(38.5)	64(54.7)
	χ2 = 1.600, P = 0.206		χ2 = 0.320, P = 0.572		χ2 = 8.722, P = 0.003*	
Insufficient fruit/ vegetable	24(10.7)	24(14.3)	15(10.3)	33(13.4)	32(11.6)	16(13.7)
	χ2 = 1.140, P = 0.286		χ2 = 0.841, P = 0.359		χ2 = 0.318, P = 0.573	
Insufficient seafood	74(33.0)	73(43.5)	62(42.5)	85(34.6)	105(38.2)	42(35.9)
	χ2 = 4.444, P = 0.035*		χ2 = 2.448, P = 0.118		χ2 = 0.183, P = 0.669	
Fast food consumption	192(85.7)	137(81.5)	121(82.9)	208(84.6)	221(80.4)	108(92.3)
	χ2 = 1.236, P = 0.266		χ2 = 0.191, P = 0.662		χ2 = 8.681, P = 0.003*	

A total of 194 females (86.6%) and 206 (83.7%) students in their third and above academic years reported insufficient water consumption compared to 121 (72.0%) males and 109 (74.7%) students in the first and second years, *P* < 0.01 and *P* = 0.029, respectively. A total of 174 (77.7%) females and 94 (80.3%) pre-obese/obese students tend to skip meals compared to 107 (63.7%) males and 187 (68.0%) normal-weight students, *P* = 0.002 and *P* = 0.013, respectively. A total of 64 (54.7%) pre-obese/obese students also reported a higher prevalence of night-eating habits compared to 106 (38.5%) normal weight students, *P* = 0.003. Insufficient seafood consumption was higher among 73 (43.5%) males compared to 74 (33.0%) females, *P* = 0.035, while fast food consumption was higher among 108 (92.3%) obese students compared to 221 (80.4%) normal-weight students, *P* = 0.003 (Table 2).

After conducting logistic regression analyses (Table 3), female gender was a significant factor associated with insufficient water intake (β = -1.02, adjusted [adj.] odds ratio [OR] = 0.3) and skipping meals (β = -0.75, adj.OR = 0.5) more than male gender, adj.P < 0.001 and adj.P = 0.001, respectively. On the other hand, male students were significantly more likely to report irregular meal times (β = 0.425, adj.OR = 1.5) and insufficient seafood consumption (β = 0.55, adj.OR = 1.7) compared to female students, adj.P = 0.046 and adj.P = 0.012, respectively. Students in their third and above years (β = 0.857, adj.OR = 2.2) reported insufficient water intake compared to students in their first and second years, adj.P = 0.003. Obesity in students was a significantly associated factor with fast food consumption (β = 0.48, adj.OR = 1.8), night-eating habits (β = 0.27, adj.OR = 1.3) and skipping meals (β = 0.41, adj.OR = 1.5) compared to normal weight students, adj.P = 0.002, adj.P = 0.004 and adj.P = 0.003, respectively.

**Table 3:** Factors associated with unhealthy dietary habits with 95% confidence interval *Statistically significant at *P *<0.05; t = Student’s t-test; 0 = reference group, 1 = compared group, β = coefficient of determination, OR = odds ratio, adj = adjusted, CI = confidence interval

	Gender Females^0^ ; Males^1^			Academic year 1^ST^ & 2^ND^ ^0^; 3^RD^ & above^1^			BMI (category) Under/Normal^0^;Pre-obese/obese^1^		
	β	adj.OR	[95%CI]	β	adj.OR	[95%CI]	β	adj.OR	[95%CI]
Sweetened beverages	0.119	1.1	[0.7-1.7]	-0.084	0.9	[0.6-1.4]	0.160	1.2	[1-1.4]
	adj.P = 0.585			adj.P = 0.706			adj.P = 0.120		
Insufficient hydration	-1.058	0.3	[0.2-0.6]	0.857	2.2	[1.3-3.8]	0.001	0.9	[0.7-1.2]
	adj.P<0.001*			adj.P = 0.003*			adj.P = 0.399		
Drinking energy drinks	0.162	1.2	[0.7-2.1]	-0.170	0.8	[0.5-1.5]	0.018	1.0	[0.7-1.3]
	adj.P = 0.572			adj.P=0.555			adj.P = 0.999		
Irregular meal times	0.425	1.5	[1.0-2.3]	-0.118	0.6	[0.6-1.4]	0.272	0.9	[0.8-1.1
	adj.P = 0.046*			adj.P = 0.588			adj.P = 0.386		
Snacks between meals	-0.368	0.7	[0.4-1.1]	-0.437	0.8	[0.8-1.1]	-0.057	1.0	[0.8-1.3]
	adj.P = 0.146			adj.P = 0.109			adj.P = 0.895		
Skipping meals	-0.755	0.5	[0.3-0.8]	-0.271	0.7	[0.5-1.2]	0.160	1.5	[1.2-1.9]
	adj.P = 0.001*			adj.P = 0.274			adj.P = 0.003*		
Night eating habit	0.208	1.2	[0.81-1.9]	-0.225	0.8	[0.5-1.2]	0.001	1.3	[1.1-1.6]
	adj.P = 0.330			adj.P = 0.305			adj.P = 0.004*		
Insufficient fruit/ vegetable	0.256	1.3	[0.7-2.4]	0.263	0.6	[0.7-2.5]	0.018	1.09	[0.8-1.6]
	adj.P = 0.420			adj.P = 0.438			adj.P = 0.554		
Insufficient seafood	0.553	1.7	[1.1-2.7]	-0.419	0.8	[0.4-1.0]	0.272	1	[0.8-1.2]
	adj.P = 0.012*			adj.P=0.058			adj.P = 0.580		
Fast food consumption	-0.490	0.6	[0.4-1.1]	0.106	0.7	[0.6-2]	-0.057	1.8	[1.2-2.6]
	adj.P = 0.089			adj.P=0.717			adj.P = 0.002*		

## Discussion

Students enrolled in faculties of health sciences are expected to be more cautious about their diet, as their knowledge sought from the academic curricula should reflect primarily on their dietary habits. These students are the future physicians, pharmacists, nurses or other allied health care specialists, who are pictured as role models to their patients, families, and community. Readers might debate the nature of the relationship between the quality of dietary habits and academic performance, as an altered diet might not directly affect the academic performance. Numerous mediating or contributing factors might also influence academic performance, such as physical activity, sleep hygiene, and methods of teaching/learning. It is also speculated that poor academic performance might alter the dietary habit, due to stress or depression. The primary message to college students is that adherence to a healthy dietary habit is not a pathway to better academic performance, but rather to promote their personal health and to exemplify themselves as influential role models within their community. 

The consumption levels of drinking sweetened beverages have increased three folds in the past two decades, and regular daily consumption has been associated with multiple health problems such as diabetes, tooth decay and heart diseases [14]. The relationship between sugars in soft drinks and health has been examined in the context of metabolic disorders, yet experimental evidence in human and animals suggested that high-fructose corn syrup and sucrose found in soft drinks resulted in adverse effects on the hippocampus in the brain that is responsible for learning and memorizing [15].

The academic performance of American students who drink energy beverages at least once weekly has been reported to be lower than those who didn’t [16]. Side effects of energy drinks such as headaches and heart palpitations have been reported widely in literature, and this phenomenon is becoming more prevalent among young adults [17]. Students usually engage in energy drink consumption to compensate for sleep deprivation or to gain energy [18]. Therefore it is important to correct the misconception among college students that by resorting to energy beverages, their academic performance will improve. Lower gastrointestinal digested foods such as well balanced meals (especially breakfasts) provide students with a more sustained energy release during the day, thus creating a better glucose environment for the brain and energy for the body.

Fish carries many health benefits, such as omega-3 long-chain polyunsaturated fatty acids that are not only important for cardiovascular health but to the many aspects of brain functioning such as neuronal membrane fluidity, neurotransmission, signal transduction and blood-brain barrier functions [19]. Two studies have acknowledged the fact that omega-3 intake had significantly improved academic and cognitive performance [20,21]. Therefore, college students ought to consume a diverse diet of proteins, with specific attention to at least 227 g seafood on a weekly bases [13]. 

Gender differences were observed with regard to insufficient hydration, irregular meal times, skipping meals, and insufficient seafood. Studies have shown that females tend to consume more fruits/vegetables, legumes, and sweets/cakes in comparison to males [22]. Males tend to consume fish or food richer in fat, such as meat and soft/carbonated drinks [22]. Generally, females tend to invest in matters related to food quality/quantity; therefore, they have a better knowledge of nutrition and weight loss measures [23]. Thus, the eating behaviors of females and males will be different. Females are more likely to eat low-calorie foods and evade overeating by skipping meals, which was similar to findings in this setting [24].

Obesity was associated with skipping meals, night eating habits, and fast food consumption. This logic finding was similar to findings reported in the literature, as a night-eating habit was classified as a dysfunction of the circadian rhythm, a disassociation between eating and sleeping. This unhealthy dietary habit is characterized by a lack of desire to eat breakfast, a strong urge to eat between dinner and sleep or during the night, and linking eating to sleeping [25]. Numerous studies have proven an association between night eating habits and obesity [25]. Inconsistent results were present when exploring gender differences with night eating habits [26]. In addition, the timing of meals is related to obesity, and skipping breakfast significantly influences both waist circumference and BMI [27]. One study concluded that eating breakfast > 4 times per week prevents excessive body weight gain in comparison to skipping breakfast in both genders [28]. Similar types of results were found in our present study; fast food consumption has been reported to be higher among younger adults (mostly men), and it was strongly associated with obesity [29].

Few limitations have been encountered in this study. The nature of the retrospective design entails that there is a slight chance of recall bias in terms of detailed dietary habits. Dietary habits might not be consistent with time, so statements were focused on short time intervals, i.e. daily or weekly, to overcome these recall biases. Other unhealthy dietary habits haven’t been questioned in this study, such as consumption of desserts, salt intake, coffee intake, and alcohol consumption. Findings in this study can be generalized to other student populations, as the analyzed sample is representative of a major university that encompasses several faculties of health sciences. 

Recommendations

All students, whether pursuing a health-related or non-health related specialty, are advised to adopt healthy dietary habits and encourage their peers to do so. For college administrators, a set of environmental modifications may influence students and boost their compliance with healthy diets. These include posting flyers/posters, presenting awareness seminars, and campaigns throughout the academic year. Labeling cafeteria meals with brief instructions on the nutritional values/calories would visually remind students of what they are buying and consuming. Vending machines within the college premises could provide healthier snacks too. At the governmental levels, firm verdicts are required to elevate taxation on sugar-sweetened beverages and energy drinks, to favor the purchase of better healthy drinks such as fresh juices and shakes.

## Conclusions

This present study revealed that the compliance of college students enrolled in health science faculties with healthy dietary habits was less than optimal. These students are the future role models within their community and should be more vigilant about their dietary lifestyle. Gender differences were observed in regards to insufficient hydration, skipping of meals and insufficient seafood consumption. Obesity in college students was associated with skipping meals, night-eating habits, and fast food consumption.
